# Artificial intelligence in Brazilian public health: potential, challenges, and ethical implications for the Brazilian Unified Health System

**DOI:** 10.1590/1980-549720260006

**Published:** 2026-03-16

**Authors:** Marcela Quaresma Soares, Alexandre Dias Porto Chiavegatto

**Affiliations:** I Universidade de São Paulo, School of Public Health - São Paulo (SP), Brazil.

**Keywords:** Artificial intelligence, Public Health, Unified Health System, Health equity, Health ethics

## Abstract

**Objectives:**

To critically analyze the potential, challenges, and ethical implications of incorporating artificial intelligence (AI) into Brazilian public health, in light of the principles of the Unified Health System (SUS), also considering its interfaces with epidemiological practice.

**Methods:**

This is a theoretical-analytical paper based on national and international literature, which articulates core AI concepts with political-epistemological reflections from Public Health. The approach includes discussions on machine learning, deep learning, natural language processing, and large language models, focusing on applications within the SUS context.

**Results:**

Multiple opportunities for using AI to strengthen the SUS are identified, including prediction of health events, diagnostic support, service regulation, and public policy development. However, structural barriers such as fragmented information systems, regional inequalities, and gaps in professional training are highlighted. Issues such as algorithmic fairness, explainability, technological sovereignty, and digital literacy emerge as key dimensions for the responsible adoption of AI.

**Conclusion:**

AI is not neutral, and its integration into the SUS must be guided by democratic principles and sensitivity to social vulnerabilities, or it risks reinforcing technocratic and exclusionary models. The struggle over the meaning of innovation is, therefore, also a struggle over the future of public health in Brazil.

## INTRODUCTION

Artificial Intelligence (AI) has emerged as a promising technology for addressing contemporary challenges in public health. Its applications encompass support for clinical diagnosis, epidemiological surveillance, and the development of policies informed by large and complex datasets^1^ ^,^ ^2^ ^,^ ^3^. Despite this potential, its incorporation requires strategic attention to structural inequalities, health system limitations, and the ethical and social risks associated with the intensive use of data^4^ ^,^ ^5^.

In Brazil, the incorporation of AI is unfolding in a context still marked by regional asymmetries, fragmented information systems, and unequal access to technological infrastructure. Within the Brazilian Unified Health System (*Sistema Único de Saúde* - SUS), the trajectory of digital health has advanced, albeit unevenly. Examples include the *e-SUS Atenção Primária à Saúde* (e-SUS APS) strategy, designed to support the computerization of Primary Health Care; *Meu SUS Digital* (formerly *Conecte SUS*), which aims to expand citizens’ access to their clinical information; and *e-SUS Linha da Vida*, currently in the implementation phase, which seeks to integrate health surveillance data. Although these initiatives are at different stages of maturity, they collectively indicate an ongoing transition in public health management, with increasing emphasis on real-time information and interoperability across systems^6^ ^,^ ^7^ ^,^ ^8^.

This movement is aligned with the Digital Health Strategy for Brazil 2020-2028 (*Estratégia de Saúde Digital para o Brasil 2020-2028*)^9^ and AI for the Good of All: Brazilian Artificial Intelligence Plan (*IA para o Bem de Todos: Plano Brasileiro de Inteligência Artificial*)^10^, both of which aim to consolidate an integrated public data ecosystem capable of supporting the incorporation of emerging technologies, such as AI. However, the absence of specific guidelines for its responsible adoption may compromise progress and exacerbate existing inequalities.

In this context, the present essay aimed to critically examine the potential, challenges, and ethical implications associated with the incorporation of AI into Brazilian public health, in alignment with the principles of SUS and with attention to its interfaces with epidemiological practice.

## METHODS

This theoretical-analytical essay is grounded in national and international literature and articulates core concepts of AI with political-epistemological reflections on Public Health. The approach encompasses discussions on machine learning, deep learning, natural language processing, and large language models, with emphasis on their applications within SUS.

As a basis for developing the arguments, academic publications, official documents, technical reports from international organizations, and reference works on AI and public health were consulted. The materials were selected with emphasis on recent publications, prioritizing sources that addressed the application of AI in the public sector, particularly within SUS. The text was organized according to an analytical framework structured around the potential, challenges, and ethical implications of AI.

## Data availability statement

This statement does not apply, as the study did not use empirical data.

## RESULTS

For purposes of presentation and analytical clarity, the results of this essay are organized into four thematic areas:

1. Foundations and key concepts of artificial intelligence in public health;
2. Potential applications of AI in the context of SUS;
3. Structural, technical, and regulatory challenges for implementing AI in SUS; and
4. Equity and Algorithmic Fairness: Ethical Challenges in the Brazilian Context. This structure aims to organize the proposed critical reflection without establishing a rigid hierarchy among the topics.

### Fundamentals and key concepts of artificial intelligence in public health

AI differs from traditional automation systems through its capacity to learn from and adapt to new data. Its growing relevance in public health is closely linked to the phenomenon of big data, characterized by the large volume, variety, and velocity of information generation, as well as the need to ensure its veracity and analytical value^3^ ^,^ ^11^. Within SUS, these data sources include epidemiological surveillance records, administrative databases, electronic medical records, and medication dispensing systems, along with emerging inputs such as mobile devices and social media. This extensive information landscape constitutes the essential raw material for the development of AI systems^1^ ^,^ ^6^.

Among the main definitions in the field, the following stand out: machine learning (ML), which focuses on identifying patterns and predicting events, such as outbreaks or hospital readmissions; deep learning (DL), which uses neural networks to process complex data, including medical images; natural language processing (NLP), which enables the extraction of information from texts and electronic medical records; and large language models (LLMs), which enhance the capacity to generate and summarize language with a high degree of sophistication^1^ ^,^ ^2^ ^,^ ^12^ ^,^ ^13^ ^,^ ^14^ ^,^ ^15^ ^,^ ^16^.

**Figure 1.**
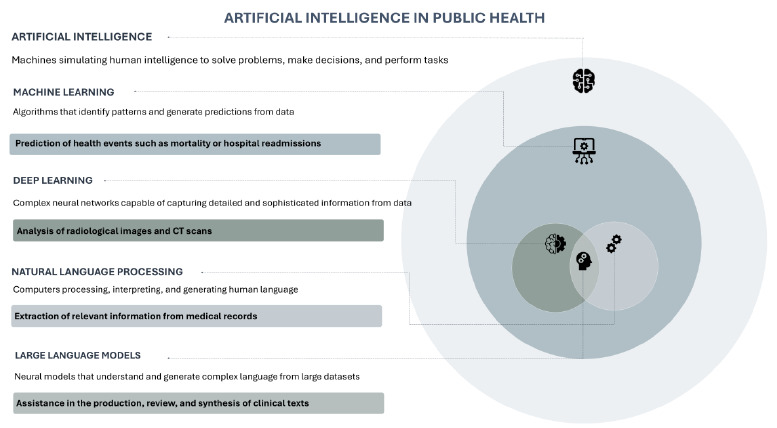
Conceptual framework of artificial intelligence in public health.

Note: Illustrated infographic for didactic purposes, intended to highlight conceptual differences between artificial intelligence approaches, without representing strict hierarchies or boundaries between areas. Source: Authors’ elaboration, using icons from Freepik.

The main techniques and illustrative applications are summarized in Figure 1, which presents the concepts discussed in this section in a didactic manner.

### Potential of artificial intelligence in the context of the Brazilian Unified Health System

The literature identifies several ways in which AI can contribute to strengthening SUS, particularly in areas where large-scale data analysis can enhance the system’s responsiveness^17^ ^,^ ^18^.

One of the main fields of application is *epidemiological surveillance*, with algorithms designed for the early detection of outbreaks and adverse events based on the real-time analysis of syndromic data, administrative records, news, and digital media. Such applications have been explored in contexts including the surveillance of dengue fever, COVID-19, and severe acute respiratory syndrome (SARS), demonstrating potential for anticipating public health responses^19^ ^,^ ^20^ ^,^ ^21^. In addition to enhancing monitoring capacity, AI can facilitate the integration of multiple, traditionally fragmented data sources and generate information that supports timely decision-making.

In this context, the concept of “epidemiological intelligence”^22^ gains prominence, as it synthesizes the articulation between automated processing of large volumes of data and strategic responses to collective health needs. Documented experiences also highlight the potential use of AI in early warning systems for epidemics, integrating structured and unstructured information in an automated manner^13^ ^,^ ^23^. Recent advances, such as the use of generative models for prediction and scenario simulation^24^, further reinforce the feasibility of applying AI in early warning systems and epidemiological planning.

Beyond detection, AI strengthens predictive modeling of outbreaks. ML models can, for example, integrate arbovirus notification data with climatic, demographic, and mobility variables to generate local-level predictions of outbreak probability, thereby supporting vector control strategies. Another frontier of epidemiological application involves improving information systems through NLP techniques to analyze the textual fields of death certificates, contributing to the reduction of ill-defined causes and the identification of comorbidity patterns not captured by traditional coding methods^25^.

A recent example of the potential of these technologies is the use of generative models capable of learning the natural history of human diseases from health records. These systems can predict multiple conditions simultaneously and generate synthetic health trajectories to support epidemiological planning and precision medicine. Despite these advances, this remains an emerging frontier that requires further refinement and validation before large-scale practical application^24^.

Examples of AI applications in epidemiological surveillance in Brazil are summarized in Table 1, illustrating their practical applicability.

**Table 1.** Applications of Artificial Intelligence in Epidemiological Surveillance and their benefits for the Brazilian Unified Health System.

Application area	Examples of AI use	Potential benefits for SUS
Surveillance of health events	Prediction of dengue, COVID-19, and severe acute respiratory syndrome cases based on syndromic and digital data.	Early outbreak detection; faster public health response.
Epidemic and outbreak modeling	Scenario simulations using generative models and machine learning.	Anticipation of scenarios; resource and intervention planning.
Mortality records	Natural language processing to qualify causes of death in the Mortality Information System.	Improved data quality and mortality surveillance.

AI: Artificial Intelligence; SUS: Brazilian Unified Health System (*Sistema Único de Saúde*).

Another strategic application of AI involves the *management of health services*. Predictive models can be used to optimize queue management, inventory control, supply logistics, and resource allocation in healthcare, thereby contributing to improvements in the user experience^2^ ^,^ ^26^ ^,^ ^27^.

AI can also contribute to expanding *access to care in remote regions* through clinical decision support tools integrated into telemedicine platforms and automated triage systems. Predictive analyses of service demand may reduce waiting lists and help prevent overload in health units^28^. In areas with shortages of specialized professionals, such as in Northern Brazil, these solutions have been explored as mechanisms to improve referral processes and reduce territorial inequalities in access to care^26^ ^,^ ^29^.

Similarly, AI models applied to *diagnostic support* in medical imaging (including X-rays, computed tomography scans, and digital pathology slides) and laboratory tests have demonstrated the potential to anticipate diagnoses and reduce errors, thereby increasing clinical accuracy^2^ ^,^ ^26^. Despite these advances, challenges persist regarding validation across diverse contexts and the safe integration of these tools into clinical practice, elements that are essential to ensuring their responsible adoption.

In the context of *personalization and precision medicine*, the integrated analysis of genomic, clinical, and contextual data through AI paves the way for more individualized and effective therapeutic approaches^30^.

Furthermore, AI can support the *formulation of evidence-based public policies* by enabling the construction of population models, the identification of health inequities, and the prioritization of actions according to epidemiological and social profiles. In contexts with limited local analytical capacity, AI can assist in extracting relevant information from clinical records, including data on social conditions associated with health, thereby strengthening the territorial planning of Health Care Networks.

**Figure 2.**
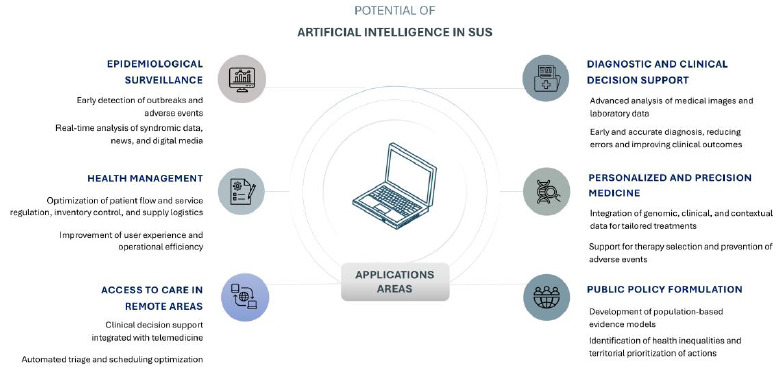
Visual summary of the contributions of artificial intelligence to strengthening the Brazilian Unified Health System.

Source: Authors’ elaboration, using icons from Freepik.

These applications demonstrate that AI, when guided by equity criteria and aligned with the needs of SUS, can serve as a strategic tool for addressing historical challenges. The main areas of AI application within the SUS are summarized in Figure 2.

### Structural, technical, and regulatory challenges for the implementation of Artificial Intelligence in the Brazilian Unified Health System

Although promising, the implementation of AI in SUS faces significant challenges. At the technical level, the main obstacles include the low quality and fragmentation of information systems, limited interoperability among platforms, and the lack of standardization in data collection^31^ ^,^ ^32^.

In the human domain, one of the central challenges is the gap in the training of healthcare professionals to work with digital technologies. Most professional categories remain insufficiently prepared to interpret predictive algorithms, understand outcome probabilities, and integrate digital tools into care delivery. This disconnect between clinical practice and the functioning of algorithmic models contributes both to resistance to their adoption and to their uncritical use in contexts where human supervision should be indispensable.

One emerging proposal is the strengthening of “algorithmic literacy” among health professionals, understood as the ability to comprehend how AI systems operate, recognize their limitations, and integrate them into clinical and public health management processes. Continuing education and technical training initiatives that incorporate basic concepts of data science, digital equity, and algorithmic governance have been identified as essential for a safe and participatory transition^17^ ^,^ ^33^. In addition, pilot experiences in Brazil have tested training modules on digital health in multiprofessional residencies and technical training programs in health surveillance, indicating possible pathways for institutionalizing this agenda^28^.

Regional inequalities represent another significant obstacle. While some urban centers possess more advanced technological infrastructure, peripheral and rural regions face basic limitations in connectivity and equipment, factors that could exacerbate longstanding disparities if AI is implemented uniformly^34^.

In the legal sphere, although the General Data Protection Law (*Lei Geral de Proteção de Dados Pessoais* - LGPD)^35^ represents an important initial milestone, normative gaps persist regarding algorithmic transparency, the explainability of automated decisions, and accountability in cases of error^31^ ^,^ ^33^. A tool recently proposed by the Pan American Health Organization (PAHO) enables countries to systematically assess their degree of institutional readiness for the adoption of AI in public health. This instrument incorporates technical, legal, ethical, and organizational dimensions and functions as a practical roadmap to guide political and operational decisions in the regulatory field^36^.

### Algorithmic Fairness: ethical challenges in the Brazilian context

Beyond technical and structural challenges, the adoption of AI in public health requires careful attention to ethical implications and to the risk of exacerbating existing inequities. Issues such as algorithmic fairness are central in a context marked by historical inequalities and institutional vulnerabilities.

The data used in AI applications are not neutral: they reflect historical human inequalities, institutional gaps, and patterns of social exclusion. When not properly contextualized, these elements may be amplified by algorithmic models, reinforcing inequities rather than mitigating them. Recent studies indicate that even state-of-the-art models reproduce biases present in their training datasets, including the “healthy volunteer bias” and patterns of missing data associated with the British biobank collection process^24^.

In this context, the debate on algorithmic fairness gains prominence, advancing mechanisms to mitigate biases and ensure that systems generate equitable decisions across different population groups^1^ ^,^ ^37^ ^,^ ^38^ ^,^ ^39^.

International initiatives have emphasized principles such as equity, transparency, explainability, and security as foundational pillars of trustworthy AI^14^ ^,^ ^40^. In the Brazilian context, marked by territorial and institutional inequalities, these principles should guide all stages of model development and assessment within SUS, taking into account the diverse population realities^4^ ^,^ ^5^.

One of the most debated issues concerns the use of sensitive variables, such as race and ethnicity. These data are essential for identifying health inequities; however, when used without proper contextualization, they can reproduce stigmas and reinforce structural discrimination. Studies recommend ethical safeguards such as continuous auditing, engagement of affected communities, and intergroup validation to mitigate these risks^37^ ^,^ ^38^ ^,^ ^41^. Models trained with foreign or generic datasets, without local validation, may produce ineffective or even harmful applications within SUS, thereby compromising the equity and effectiveness of health actions^42^.

Another relevant ethical consideration concerns explainability. Models with low transparency, often referred to as “black boxes,” limit the ability of professionals and users to understand how decisions are generated, thereby weakening social participation and institutional accountability^33^ ^,^ ^36^.

To address this issue, several guidelines have been proposed. The Transparent Reporting of a Multivariable Prediction Model for Individual Prognosis or Diagnosis - Artificial Intelligence (TRIPOD+AI) provides standards for transparency, replicability, and subgroup validation^43^. Complementarily, the Prediction Model Risk of Bias Assessment Tool - Artificial Intelligence (PROBAST+AI) offers a methodological framework for evaluating the risk of bias, applicability, and quality of studies that develop or validate predictive models using regression or AI-based techniques^44^.

Interpretability tools such as SHapley Additive exPlanations (SHAP) values have also been used to make models more understandable, particularly in clinical settings, thereby promoting informed decision-making and increasing confidence among healthcare professionals^45^.

## DISCUSSION

In Brazil, AI in public health is a rapidly expanding field, marked by disputes, potential transformations, and significant ethical challenges. Beyond generating new possibilities, these technologies can contribute decisively to improving policies, expanding access to care through automated screening in remote regions, and enhancing health surveillance by enabling real-time analysis of syndromic and population mobility data. However, their incorporation into SUS must be guided by democratic principles, sensitivity to inequalities, and a commitment to social rights.

The mere adoption of new technologies does not guarantee justice in healthcare. Beyond technical proficiency, it is necessary to contest the very meanings attributed to innovation. In this scenario, AI can serve either the commodification of healthcare or the strengthening of the public system, a choice that is not solely technical, but also political. Ensuring that AI advances equity requires a set of coordinated political and institutional actions. Among these, the strengthening of democratic governance structures is essential, including ethical and technical committees that incorporate the social participation of health councils and civil society, with the aim of transparently auditing the functioning and impacts of algorithms used in SUS^23^ ^,^ ^46^.

It is also essential to invest in data infrastructure and critical education, ensuring the quality, interoperability, and security of information while promoting algorithmic literacy so that professionals and managers can interpret, question, and apply results in a qualified manner^17^. Furthermore, fostering technological sovereignty is crucial for reducing external dependence, which requires encouraging the development of national algorithms, prioritizing open-source software, and mandating rigorous local validation before the implementation of any technology to ensure its suitability to the Brazilian epidemiological and social context^23^.

As the specialized literature indicates, the responsible adoption of AI extends beyond algorithmic effectiveness and requires reflection on who benefits, who may be excluded, and how the technology reshapes practices, relationships, and institutional structures. More important than predicting risks or supporting decisions is considering the model of society that these tools are intended to help strengthen. In the field of public health, AI must be understood as a collective construction, with implications that are not only technical but also ethical, political, and social.

Finally, when aligned with the principles of SUS and the critical tradition of public health, AI can contribute not only to efficiency gains but also to the strengthening of a public health policy that recognizes diversity, promotes social justice, and reinforces the role of the State as a guarantor of rights. This potential, however, can only be realized through deliberate political choices and the democratic governance of technological innovation.
