# Supplementary material for: Artificial intelligence in Brazilian public health: potential, challenges, and ethical implications for the Brazilian Unified Health System
Source: Rev Bras Epidemiol. 2026 Mar 16;29:e260006. doi: 10.1590/1980-549720260006 (PMC12995343; doi:10.1590/1980-549720260006)
Supplement: Translation

# Translation: Inteligência artificial na saúde pública brasileira: potencialidades, desafios e implicações éticas para o Sistema Único de Saúde

**DOI:** 10.1590/1980-549720260006.2

**Authors:** Marcela Quaresma Soares, Alexandre Dias Porto Chiavegatto

**Affiliations:** I Universidade de São Paulo, Faculdade de Saúde Pública - São Paulo (SP), Brasil.

**Keywords:** Inteligência artificial, Saúde pública, Sistema Único de Saúde, Equidade em saúde, Ética em saúde

## Abstract

**Objetivo:**

Analisar de forma crítica as potencialidades, os desafios e as implicações éticas da incorporação da Inteligência Artificial (IA) à saúde pública brasileira, à luz dos princípios do Sistema Único de Saúde (SUS), considerando também suas interfaces com a prática epidemiológica.

**Métodos:**

Trata-se de um artigo de natureza teórico-analítica, fundamentado em literatura nacional e internacional, que articula conceitos centrais da IA e reflexões político-epistemológicas da Saúde Coletiva. A abordagem contempla discussões sobre aprendizado de máquina, aprendizado profundo, processamento de linguagem natural e grandes modelos de linguagem, com foco em aplicações no contexto do SUS.

**Resultados:**

Identifica-se múltiplas possibilidades de uso da IA para o fortalecimento do SUS, incluindo predição de eventos em saúde, apoio ao diagnóstico, regulação de serviços e formulação de políticas públicas. No entanto, destacam-se barreiras estruturais, como a fragmentação dos sistemas de informação, desigualdades regionais e lacunas na formação profissional. Questões como equidade e justiça algorítmica, explicabilidade, soberania tecnológica e letramento digital emergem como dimensões essenciais para a adoção responsável da IA.

**Conclusão:**

A IA não é neutra, e sua integração ao SUS deve ser guiada por princípios democráticos e sensibilidade às vulnerabilidades sociais, sob pena de reforçar modelos tecnocráticos e excludentes. A disputa pelo sentido da inovação é, portanto, também uma disputa pelo futuro da saúde pública no Brasil.

## INTRODUÇÃO

A Inteligência Artificial (IA) tem se consolidado como uma tecnologia promissora no enfrentamento de desafios contemporâneos em saúde pública. Suas aplicações vão do suporte ao diagnóstico clínico à vigilância epidemiológica, passando pela formulação de políticas orientadas por dados massivos e complexos^1^ ^,^ ^2^ ^,^ ^3^. Apesar desse potencial, sua incorporação requer atenção estratégica às desigualdades estruturais, às limitações do sistema de saúde e aos riscos éticos e sociais associados ao uso intensivo de dados^4^ ^,^ ^5^.

No Brasil a incorporação da IA ocorre em um cenário ainda marcado por assimetrias regionais, fragmentação de sistemas de informação e desigualdades no acesso à infraestrutura tecnológica. No âmbito do Sistema Único de Saúde (SUS), a trajetória da saúde digital tem avançado, embora de forma desigual, com iniciativas como a estratégia e-SUS APS, voltada à informatização da Atenção Primária à Saúde; o Meu SUS Digital (antigo Conecte SUS), que busca ampliar o acesso do cidadão às suas informações clínicas; e o e-SUS Linha da Vida, ainda em fase de implementação, que pretende integrar dados da vigilância em saúde. Embora em diferentes estágios de maturidade, essas estratégias sinalizam uma transição em curso na gestão pública da saúde, com maior valorização da informação em tempo real e da interoperabilidade entre sistemas^6^ ^,^ ^7^ ^,^ ^8^.

Esse movimento está alinhado à Estratégia de Saúde Digital para o Brasil 2020-2028^9^ e ao IA para o Bem de Todos: Plano Brasileiro de Inteligência Artificial^10^, ambos voltados à consolidação de um ecossistema de dados públicos integrado e preparado para a incorporação de tecnologias emergentes, como a IA. No entanto, a ausência de diretrizes específicas para sua adoção responsável pode comprometer avanços e ampliar desigualdades já existentes.

Nesse contexto, o presente ensaio tem como objetivo analisar, de forma crítica, as potencialidades, os desafios e as implicações éticas da incorporação da IA à saúde pública brasileira, à luz dos princípios do SUS, considerando também suas interfaces com a prática epidemiológica.

## MÉTODOS

Trata-se de um ensaio de natureza teórico-analítica, fundamentado em literatura nacional e internacional, que articula conceitos centrais da IA e reflexões político-epistemológicas da Saúde Coletiva. A abordagem contempla discussões sobre aprendizado de máquina, aprendizado profundo, processamento de linguagem natural e grandes modelos de linguagem, com foco em aplicações no contexto do SUS.

Como base para o desenvolvimento dos argumentos, foram consultados publicações acadêmicas, documentos oficiais, relatórios técnicos de organismos internacionais e obras de referência sobre inteligência artificial e saúde pública. Os materiais foram selecionados com ênfase em produções recentes, priorizando fontes que abordassem a aplicação da IA no setor público, especialmente no SUS. A organização do texto seguiu uma lógica analítica, estruturada em torno de potencialidades, desafios e implicações éticas da IA.

## Declaração de disponibilidade de dados

Não se aplica, pois este estudo não utilizou dados empíricos

## RESULTADOS

Para fins de apresentação e objetividade analítica, os resultados deste ensaio estão organizados em quatro eixos temáticos:

1. Fundamentos e conceitos-chave da inteligência artificial na saúde pública;
2. Potencialidades da inteligência artificial no contexto do SUS;
3. Desafios estruturais, técnicos e regulatórios para a implementação da IA no SUS; e
4. Equidade e Justiça Algorítmica (*Fairness*): Desafios Éticos no Contexto Brasileiro. Essa divisão visa estruturar a reflexão crítica proposta, sem estabelecer hierarquia rígida entre os temas.

### Fundamentos e conceitos-chave da inteligência artificial na saúde pública

A IA distingue-se de sistemas de automação tradicionais por sua capacidade de aprender e adaptar-se a novos dados. Sua ascensão na saúde pública está intrinsicamente ligada ao fenômeno do *big data*, caracterizado por grande volume, variedade e velocidade de geração das informações, bem como pela necessidade de assegurar sua veracidade e seu valor analítico^3^ ^,^ ^11^. No SUS, isso inclui dados de vigilância epidemiológica, registros administrativos, prontuários eletrônicos e bases de dispensação de medicamentos, além de fontes emergentes como dispositivos móveis e mídias sociais. Essa riqueza de informações constitui a matéria-prima essencial para o desenvolvimento de sistemas de IA^1^ ^,^ ^6^.

Entre as principais definições da área, destacam-se o aprendizado de máquina (*Machine Learning* - ML), voltado à identificação de padrões e predição de eventos, como surtos ou readmissões hospitalares; o aprendizado profundo (*Deep Learning* - DL), que utiliza redes neurais para lidar com dados complexos, como imagens médicas; o processamento de linguagem natural (*Natural Language Processing* - NLP), que permite extrair informações de textos e prontuários eletrônicos; e os grandes modelos de linguagem (*Large Language Models* - LLMs), que ampliam a capacidade de gerar e resumir linguagem com alto grau de sofisticação^1^ ^,^ ^2^ ^,^ ^12^ ^,^ ^13^ ^,^ ^14^ ^,^ ^15^ ^,^ ^16^.

As principais técnicas e exemplos de aplicações estão sintetizados de forma didática na Figura 1, que ilustra os conceitos discutidos nesta seção.

**Figura 1.** Estrutura conceitual da inteligência artificial na saúde pública.

Nota: Infográfico ilustrado com fins didáticos para destacar diferenças conceituais entre abordagens da inteligência artificial, sem representar hierarquias ou limites rígidos entre as áreas. Fonte: Elaboração própria, com uso de ícones do *Freepik*.

### Potencialidades da inteligência artificial no contexto do Sistema Único de Saúde

A literatura aponta diversas formas pelas quais a IA pode contribuir para o fortalecimento do SUS, especialmente em áreas em que a análise de dados em larga escala pode ampliar a capacidade de resposta do sistema^17^ ^,^ ^18^.

Um dos principais campos de aplicação é a *vigilância epidemiológica*, com algoritmos voltados à detecção precoce de surtos e eventos adversos com base na análise de dados sindrômicos, registros administrativos, notícias e mídias digitais em tempo real. Essas aplicações já foram exploradas em situações como a vigilância de dengue, COVID-19 e Síndrome Respiratória Aguda Grave (SRAG), demonstrando potencial para antecipar respostas em saúde coletiva^19^ ^,^ ^20^ ^,^ ^21^. Além de ampliar a capacidade de monitoramento, a IA pode favorecer a integração de múltiplas fontes de dados, tradicionalmente fragmentadas, e gerar informações úteis para a tomada de decisão em tempo oportuno.

Nesse contexto, ganha relevância o conceito de “inteligência epidemiológica”^22^, que sintetiza essa articulação entre processamento automatizado de grandes volumes de dados e resposta estratégica às necessidades coletivas em saúde. Experiências já documentadas também apontam para o uso potencial de IA em sistemas de alerta precoce para epidemias, integrando informações estruturadas e não estruturadas de forma automatizada^13^ ^,^ ^23^. Avanços recentes, como o uso de modelos generativos para predição e simulação de cenários^24^, também reforçam a possibilidade de aplicação da IA em sistemas de alerta precoce e planejamento epidemiológico.

Para além da detecção, a IA potencializa a modelagem preditiva de surtos. Modelos de ML podem, por exemplo, integrar dados de notificação de arboviroses com variáveis climáticas, demográficas e de mobilidade para gerar predições da probabilidade de ocorrências de surtos em nível local, orientando ações de controle vetorial. Outra fronteira de aplicação epidemiológica está na qualificação dos sistemas de informação, por meio de técnicas de NLP para analisar os campos textuais das declarações de óbito, auxiliando na redução de causas mal definidas e na identificação de padrões de comorbidades que a codificação tradicional não captura^25^.

Um exemplo recente do potencial dessas tecnologias é o uso de modelos generativos capazes de aprender a história natural das doenças humanas com base em registros de saúde. Esses sistemas podem prever múltiplas condições simultaneamente e até gerar trajetórias de saúde sintéticas para apoiar o planejamento epidemiológico e a medicina de precisão. Apesar dos avanços, trata-se ainda de uma fronteira em desenvolvimento, que demanda aprimoramentos e validações adicionais antes de uma aplicação prática em larga escala^24^.

Exemplos de aplicações de IA em *vigilância epidemiológica* no Brasil estão resumidos na Tabela 1, evidenciando sua aplicabilidade prática.

**Tabela 1.** Aplicações da Inteligência Artificial na Vigilância Epidemiológica e seus benefícios para o Sistema Único de Saúde.

Campo de aplicação	Exemplos de uso de IA	Potenciais benefícios para o SUS
Vigilância de agravos	Predição de casos de dengue, COVID-19 e síndrome respiratória aguda grave com base em dados sindrômicos e digitais.	Detecção precoce de surtos; maior rapidez na resposta em saúde coletiva.
Modelagem de epidemias e surtos	Simulações de cenários com modelos generativos e aprendizado de máquina.	Antecipação de cenários; planejamento de recursos e intervenções.
Registros de mortalidade	Processamento de linguagem natural para qualificação das causas de óbito no Sistema de Informações sobre Mortalidade.	Melhoria da qualidade da informação e da vigilância de mortalidade.

IA: Inteligência Artificial; SUS: Sistema Único de Saúde.

Outro uso estratégico da IA diz respeito à *gestão de serviços de saúde*. Modelos preditivos podem ser aplicados para otimizar a regulação de filas, o controle de estoques, a logística de insumos e a alocação de recursos em saúde, contribuindo para melhorar a experiência do usuário^2^ ^,^ ^26^ ^,^ ^27^.

A IA pode contribuir, também, para ampliar o *acesso ao cuidado em regiões remotas*, por meio de ferramentas de apoio à decisão clínica integradas a plataformas de telemedicina e triagem automatizada. A análise preditiva da demanda por serviços pode reduzir filas de espera e evitar sobrecarga em unidades de saúde^28^. Em áreas com escassez de profissionais especializados, como no Norte do Brasil, essas soluções têm sido exploradas como forma de qualificar o encaminhamento e reduzir desigualdades territoriais no acesso ao cuidado^26^ ^,^ ^29^.

Na mesma direção, modelos de IA aplicados ao *apoio diagnóstico* em imagens médicas (como radiografias, tomografias e lâminas de patologia digital) e exames laboratoriais têm demonstrado potencial para antecipar diagnósticos e reduzir erros, aumentando a precisão clínica^2^ ^,^ ^26^. Apesar desses avanços, ainda há desafios quanto à validação em diferentes contextos e à integração segura dessas ferramentas à prática clínica, pontos essenciais para garantir sua adoção responsável.

Considerando a *personalização e a medicina de precisão*, a análise integrada de dados genômicos, clínicos e contextuais por meio de IA abre caminho para abordagens de tratamento mais personalizadas e eficazes^30^.

Além disso, a IA pode também apoiar a *formulação de políticas públicas* baseadas em evidências, permitindo a construção de modelos populacionais, a identificação de iniquidades em saúde e a priorização de ações conforme perfis epidemiológicos e sociais. Em contextos com menor capacidade analítica local, pode auxiliar na extração de informações relevantes de registros clínicos, inclusive sobre condições sociais associadas à saúde, fortalecendo o planejamento territorializado das Redes de Atenção à Saúde.

Essas aplicações demonstram que a IA, quando orientada por critérios de equidade e alinhada às necessidades do SUS, pode atuar como ferramenta estratégica para a superação de desafios históricos. As principais frentes de aplicação da IA no contexto do SUS estão sintetizadas na Figura 2.

**Figura 2.** Síntese visual das contribuições da inteligência artificial para o fortalecimento do Sistema Único de Saúde.

Fonte: Elaboração própria, com uso de ícones do Freepik.

### Desafios estruturais, técnicos e regulatórios para a implementação da Inteligência Artificial no Sistema Único de Saúde

Ainda que promissora, a implementação da IA no SUS possui desafios relevantes. No plano técnico, os principais desafios dizem respeito à baixa qualidade e à fragmentação dos sistemas de informação, à carência de interoperabilidade entre plataformas e à falta de padronização na coleta de dados^31^ ^,^ ^32^.

No campo humano, um dos principais desafios é a lacuna na formação de profissionais de saúde para lidar com tecnologias digitais. A maioria das categorias profissionais ainda carece de preparo para interpretar algoritmos preditivos, compreender a probabilidade de desfechos e integrar ferramentas digitais ao cuidado em saúde. Esse afastamento entre a prática clínica e o funcionamento dos modelos algorítmicos contribui para a resistência à sua adoção, bem como para o uso acrítico em contextos em que a supervisão humana deveria ser indispensável.

Uma proposta emergente é o fortalecimento do “letramento algorítmico” entre os profissionais de saúde, ou seja, sua capacidade de entender como os sistemas de IA funcionam, quais seus limites e de que modo podem ser incorporados ao processo clínico e à gestão em saúde coletiva. Iniciativas de educação permanente e formação técnica que incluam noções básicas de ciência de dados, equidade digital e governança algorítmica têm sido apontadas como fundamentais para uma transição segura e participativa^17^ ^,^ ^33^. Além disso, experiências-piloto no Brasil têm testado módulos formativos sobre saúde digital nas residências multiprofissionais e nos cursos de formação técnica em vigilância em saúde, indicando possíveis caminhos para institucionalizar essa agenda^28^.

As desigualdades regionais representam outro obstáculo. Enquanto alguns centros urbanos dispõem de infraestrutura tecnológica avançada, regiões periféricas e rurais enfrentam limitações básicas de conectividade e equipamentos, o que pode agravar disparidades históricas se a IA for implantada de forma homogênea^34^.

No âmbito jurídico, apesar da importância da Lei Geral de Proteção de Dados (LGPD)^35^ como marco inicial, ainda há lacunas normativas sobre transparência algorítmica, explicabilidade de decisões automatizadas e responsabilização em casos de erro^31^ ^,^ ^33^. Uma ferramenta recentemente proposta pela Organização Pan-Americana da Saúde (OPAS) permite que países avaliem de forma sistemática seu grau de prontidão institucional para a adoção da IA em saúde pública. O instrumento considera aspectos técnicos, jurídicos, éticos e organizacionais, servindo como um roteiro prático para orientar decisões políticas e operacionais no campo da regulação^36^.

### Equidade e justiça algorítmica (Fairness): desafios éticos no contexto brasileiro

Para além dos desafios técnicos e estruturais, a adoção da IA em saúde pública demanda atenção às implicações éticas e aos riscos de aprofundamento das iniquidades existentes. Questões como equidade e justiça algorítmica (*fairness*) ^1^ são centrais em um cenário marcado por desigualdades históricas e fragilidades institucionais.

Os dados utilizados em aplicações de IA não são neutros: eles carregam as marcas de desigualdades humanas históricas, lacunas institucionais e padrões de exclusão social. Quando não adequadamente contextualizados, esses elementos podem ser amplificados por modelos algorítmicos, reforçando iniquidades em vez de corrigi-las. Estudos recentes mostram que mesmo modelos de última geração reproduzem vieses de suas bases de treinamento, como o “viés do voluntário saudável” e padrões de ausência de dados específicos do processo de coleta do biobanco britânico^24^.

Nesse contexto, ganha destaque o debate sobre equidade e justiça algorítmica (*fairness*), que propõe mecanismos para mitigar vieses e garantir que os sistemas produzam decisões equitativas entre diferentes grupos populacionais^1^ ^,^ ^37^ ^,^ ^38^ ^,^ ^39^.

Iniciativas internacionais têm destacado princípios como equidade, transparência, explicabilidade e segurança como pilares para uma IA confiável^14^ ^,^ ^40^. No contexto brasileiro, marcado por desigualdades territoriais e institucionais, esses princípios devem orientar desde o desenho até a avaliação dos modelos aplicados ao SUS, considerando as múltiplas realidades populacionais^4^ ^,^ ^5^.

Um dos temas mais debatidos envolve o uso de variáveis sensíveis, como raça e etnia. Esses dados são fundamentais para revelar iniquidades em saúde, mas seu uso sem contextualização pode reproduzir estigmas e reforçar discriminações estruturais. Estudos recomendam salvaguardas éticas como auditorias contínuas, envolvimento das comunidades afetadas e validação intergrupos para mitigar esses riscos^37^ ^,^ ^38^ ^,^ ^41^. Modelos treinados com dados estrangeiros ou genéricos, sem validação local, podem gerar aplicações ineficazes ou prejudiciais ao SUS, comprometendo a equidade e a efetividade das ações em saúde^42^.

Outro ponto ético relevante refere-se à explicabilidade. Modelos com baixa transparência, frequentemente chamados de “caixas-pretas”, reduzem a capacidade dos profissionais e dos usuários de compreender como decisões foram tomadas, fragilizando o controle social e a responsabilização institucional^33^ ^,^ ^36^.

Para enfrentar esse problema, vêm sendo propostas diretrizes, como o *Transparent Reporting of a multivariable prediction model for Individual Prognosis Or Diagnosis - Artificial Intelligence* (TRIPOD+AI), que estabelecem padrões de transparência, replicabilidade e validação por subgrupos^43^. Complementarmente, o *Prediction Model Risk of Bias Assessment Tool AI* (PROBAST+AI) oferece uma estrutura metodológica para avaliar o risco de viés, a aplicabilidade e a qualidade dos estudos que desenvolvem ou validam modelos preditivos com técnicas de regressão ou IA^44^.

Ferramentas de interpretabilidade como os valores de SHAP (*SHapley Additive exPlanations*) também vêm sendo utilizadas para tornar os modelos mais compreensíveis, especialmente em ambientes clínicos, favorecendo decisões informadas e a confiança dos profissionais de saúde^45^.

## DISCUSSÃO

No Brasil, a IA na saúde pública se apresenta como um campo em expansão, repleto de disputas, potenciais transformações e desafios éticos relevantes. Além de gerar novas possibilidades, essas tecnologias podem contribuir de maneira decisiva para qualificar políticas, ampliar o acesso ao cuidado por meio de triagens automatizadas em regiões remotas e aprimorar a vigilância em saúde com análises em tempo real de dados sindrômicos e de mobilidade populacional. No entanto, sua incorporação ao SUS deve ser orientada por princípios democráticos, sensibilidade às desigualdades e compromisso com os direitos sociais.

A simples adoção de novas tecnologias não garante justiça em saúde. Mais do que dominar ferramentas, é necessário disputar os sentidos da inovação. Nesse cenário, a IA tanto pode servir à mercantilização da saúde quanto ao fortalecimento do sistema público, e essa escolha não é apenas técnica, mas política. Garantir que a IA esteja a serviço da equidade requer um conjunto de ações políticas e institucionais. Entre elas, destaca-se o fortalecimento de instâncias de governança democrática, com comitês éticos e técnicos que incluam a participação social de conselhos de saúde e da sociedade civil, a fim de auditar de forma transparente o funcionamento e os impactos dos algoritmos utilizados no SUS^23^ ^,^ ^46^.

Também é essencial investir em infraestrutura de dados e em educação crítica, assegurando a qualidade, interoperabilidade e segurança das informações, ao mesmo tempo que se promove o letramento algorítmico para que profissionais e gestores possam interpretar, questionar e utilizar os resultados de maneira qualificada^17^. Além disso, o fomento à soberania tecnológica é decisivo para reduzir a dependência externa, o que implica incentivar o desenvolvimento de algoritmos nacionais, priorizar *softwares* de código aberto e exigir validação local rigorosa antes da implementação de qualquer tecnologia, de modo a garantir sua adequação ao contexto epidemiológico e social brasileiro^23^.

Como aponta a literatura especializada, a adoção responsável da IA vai além da eficácia dos algoritmos e envolve refletir sobre quem são os beneficiários, quem pode ser excluído e de que forma a tecnologia transforma práticas, relações e estruturas institucionais. Mais importante do que predizer riscos ou apoiar decisões, é refletir sobre o modelo de sociedade que se pretende fortalecer por meio dessas ferramentas. A inteligência artificial, no campo da saúde pública, deve ser compreendida como uma construção coletiva, com implicações não apenas técnicas, mas também éticas, políticas e sociais.

Por fim, quando alinhada aos princípios do SUS e à tradição crítica da saúde coletiva, a IA pode contribuir não apenas para ganhos de eficiência, mas para o fortalecimento de uma política pública de saúde que reconheça a diversidade, promova justiça social e reforce o papel do Estado como garantidor de direitos. Esse potencial, no entanto, só se concretiza por meio de escolhas políticas deliberadas e de uma governança democrática da inovação tecnológica.

## Footnotes

1
 Neste artigo, o termo *fairness* é traduzido como “equidade e justiça algorítmica”, de forma a abarcar tanto as dimensões técnicas de desempenho equânime entre grupos populacionais quanto os aspectos éticos, normativos e políticos relacionados à não discriminação e à legitimidade das decisões automatizadas.
